# Supplementary material for: Impact of severe mental illnesses on health-related quality of life among patients attending the Institute of Psychiatry, Rawalpindi from 2019 to 2021: A cross-sectional study
Source: PLoS One. 2023 Aug 3;18(8):e0289080. doi: 10.1371/journal.pone.0289080 (PMC10399870; doi:10.1371/journal.pone.0289080)
Supplement: Attachment — Submitted filename: PLOS feedback .docx [file pone.0289080.s001.docx]

This study has been done well. This study could have focused on understanding the Quality of life of individuals in depth instead of only profiling clients with regard to sociodemographic. Qualitative data could have enhanced the results from the survey and quantitative data which also would have given the reader key insights.
