# Supplementary material for: Impact of severe mental illnesses on health-related quality of life among patients attending the Institute of Psychiatry, Rawalpindi from 2019 to 2021: A cross-sectional study
Source: PLoS One. 2023 Aug 3;18(8):e0289080. doi: 10.1371/journal.pone.0289080 (PMC10399870; doi:10.1371/journal.pone.0289080)
Supplement: Attachment — Submitted filename: author response to reviewers.docx [file pone.0289080.s003.docx]

**REBUTTAL LETTER**

**Thank you for all reviewers for their useful comments and suggestions, made for improving the original manuscript. we hope our attempt to deal with reviewer’s concerns is considered satisfactory.**

**For a better understanding we have colored all the clarifications in red**

PONE-D-23-01479

Impact of severe mental illnesses on health-related quality of life in patients visiting institute of psychiatry, Rawalpindi during 2019 to 2021, a cross sectional study

PLOS ONE

Review Comments to the Author

Please use the space provided to explain your answers to the questions above. You may also include additional comments for the author, including concerns about dual publication, research ethics, or publication ethics. (Please upload your review as an attachment if it exceeds 20,000 characters)

Reviewer #1: General
This is an interesting descriptive study. The analysis, while basic, is appropriate. More sophisticated analyses looking at predictors of health-related quality of life could have been undertaken, but as it stands this provides a descriptive look at health-related quality of life in patients with SMI in Pakistan. In places, the sentences do not have full stops, and the phrasing is unclear. It would benefit from a careful proof-reading. I felt the conclusion in the final paragraph were not fully supported by the results of the study – without providing comparison to the general population.
• References: These are not fully formatted, some are missing titles and journal names, and some reference numbers in the text do not match the references in the reference list (e.g. reference 13 as noted below).
• Author contributions are only listed for the first author and it is therefore unclear what the other two authors contributed.

Introduction:
• Reference 2 would be better linked to where the FDA define this, given it’s a quote, rather than to the protocol for this study.

Reference is linked to where the FDA Definition is given.

• This sentence needs to be split into the two separate points – what quality of life is, and the evidence for it. Is there a reference for recent evidence? “Hence quality of life is a broader concept, involving not only the subjective burden but also includes all aspects of daily living, interaction with others, and recent evidence and evidence on health-related quality of life is increasing and is being regarded as a major patient outcome”.

Sentence split into half.

• The references are not ordered numerically in the text, instead going reference 1, 2, 3, 15, 13, 4 etc.

References arranged according to numbers.

• Eq 5d 5l should be explained at least briefly or spelled out in the introduction.

EQ5D5L instrument is spelled out is in the introduction section and detailed description of all the components is given in the methodology section.

• Reference 13 is the ICD-10 classification, but the text refers to reference 13 as a 2018 study on health-related quality of life in people with schizophrenia. Please check all the references are correctly labelled.

Reference correctly labelled

Methods:
• Could the authors provide a reference for WHO Steps – it wasn’t clear what this is – is it an IT solution for recording data?

Reference for WHO STEPS V 3.2 given. it is a standardized tool used for collecting demographic information of the study participants and has been used in various studies. WHO STEPS includes software and supporting materials to to undertake data collection such as android devices, this helps to reduce errors, any missed data and also allows remote data submission. Additionally, also has benefit of reduced time and minimizes dependency on paper.

• And a reference for the EQ-5D-5L?

Reference for EQ5D 5L given.

• Under data collection could you detail how the data was collected during the study? Was this face-to-face interview, or self-completed questionnaire for example?

Data collected using self-completed EQ5D 5L questionnaire for health-related quality of life , there were no face to face interviews

Results:
• P=0.000, should be written as p<0.001 as it is just a rounding up ion the statistical software and p unlikely represents 0.

P value corrected

• “Eq vas scores were lower in female (49.43±27.72)” Is this mean and SD? If so, they should be labelled as such.
• Table 2: Age and marital status are also associated with EQVAS in your results, but you don’t make mention of them in your results or discussion section – why is that? Would also be useful to discuss why perhaps you don’t find an association with income, as you would perhaps expect that there would be one.
• “When eq vas was analyzed across SMI (severe mental illnesses), the Eqvas score was lower for patients suffering from depression across all demographic factors as compared to the patients suffering from schizophrenia and bipolar affective (table 3)”. But there were some interesting differences – yes, in general eqvas was lowest in those with depression, but for people over age 65 or only completing primary education it was lowest in those with schizophrenia. People with bipolar disorder have consistently the highest scores though, which is interesting and worth noting.

Additional Findings noted by the reviewers incorporated in manuscripts result section.

• Is it possible to have table 1 broken down by diagnosis as a supplementary table, or numbers included in table 3? I just wonder if there are different age profiles and other demographic differences between the three diagnoses?
• Table 3: Please label as mean and SD.

Mean and SD labelled.

• Table 3: Is there a reason there is no significance testing for this table?

There was no significance testing in this table because it shows demographic distribution of the population associated with SMI Diagnosis using mean and standard deviation, that were given against every diagnosis of SMI according to demographic distribution. significance using P value was not found.

• Table 4: Could you add % for the total SMI please?

% for the total SMI added

• Table 4: Is “unable to” a non-response?

Unable to in this table for (unable to move, or unable to do self-care etc.) corrected in the table in manuscript.

• I think it’s really interesting how well the bipolar disorder cohort report their quality of life: for self-care and activity particularly. Also interesting that people with schizophrenia have the highest amount of moderate/severe problems of self-care. Also, interesting how many of the patients with depression have mobility issues – is there a reason this is so high? I think these points could be discussed more in the discussion.

Points regarding mobility issues of patients suffering from depression discussed in the discussion that people suffering from depression have mobility issues owing to the physical symptoms of depression including insomnia, decrease appetite etc. Owing to the severity and long-term nature of illness, patients with schizophrenia have highest number of moderate/ severe problems of self-care.

Discussion:
• “Our study identified that health related quality of life, which is major patient outcome was lowered in all SMI (severe mental illness) population” – lower compared to what? You do not make a comparison in your study. Is there a reference “normal range”? The conclusion also supports the need for more intervention on health-related quality of life for those with SMI. While I agree, I am not sure that this study provides any evidence for that without comparing scores to a population without SMI. If there is a range or other study in those without SMI that you were comparing these results to, then you should include them to strengthen this point.

There was no comparison of SMI Population with any reference or normal range, comparison in between the SMI Population were made , but the comparison with any reference or normal population was beyond the scope of the study and mentioned in the limitations section

• “hence the factor of poor socio-economic status becomes more relevant to contributing to lower health related quality of life in our population.” – but you didn’t find any difference by income? Could you discuss how your findings fit into this statement?

This point removed from the discussion section.

• “another factor that can be postulated is that patients suffering from depression have preserved insight in to their illness and are more aware on effect of illness on their quality of life as compared to patients suffering from bipolar affective disorder especially with psychotic features and schizophrenia” Do the authors think this explains the difference for their cohort, given they were only looking at depression with psychotic features and therefore insight might also be less in this group?

Yes, the cohort included in this study were also patients suffering from depression with psychotic factors and insight might also be less in this group, this suggestion added in the discussion section as suggested by the reviewer

• “Another study a study conducted under STAR D trial showed that demographic factors associated with lowered health related quality of life in depression, including low education, socioeconomic status, and poor social support, and people who are widowed or divorced or separated [17]” The authors could expand on this to include their findings re marital status which is in the table but not reported in the text.

Finding of lower health related quality of life depicted in our study among people who are widowed, divorced, or separated added both in discussion and results section as suggested by the reviewer.

• “Another limitation of our study was that as our study design was correlational, the demographic factors that have impact on health-related quality of life were studied to determine any long term or causal relationship between them.” I am not sure what this means? Do you mean you weren’t looking for long term or causal relationships?

Yes, long term or causal relationships were beyond the scope of the study and added in limitation section.

• Discussion of broader limitations would also be useful – how representative is this of patients in Pakistan do you think? What could have biased your findings? These are only descriptive so perhaps the different demographic characteristics in those with depression vs schizophrenia or bipolar disorder could have affected your results? How good a tool is the EQ 5D-5L? and why was this the most appropriate for the study? Are there other tools that would have strengthened the findings? Did everyone answer all the questions, and could this have introduced bias? Could the way the data was collected influence how people responded?

Discussion of broader limitation added in the manuscript that the findings of the study cannot be generalized to Pakistani population as study carried out at tertiary care setting that is not representative to the whole Pakistani population, and other characteristics could also have affected the results, these limitations have been added in the manuscript.

EQ5D 5L was the only instrument used in our study and no comparison was made between this instrument and other instruments used to assess the quality of life in our population.

Reviewer #2: This study has been done well. This study could have focused on understanding the Quality of life of individuals in depth instead of only profiling clients regarding sociodemographic. Qualitative data could have enhanced the results from the survey and quantitative data which also would have given the reader key insights.

Reviewer #3: Thank you for giving me the opportunity to review this interesting article.
In general, the content of the article is appropriate, but the writing should be improved.
I have carefully reviewed the content of each section and provided detailed comments. Additionally, I have taken the liberty of offering suggestions to improve the writing, which I believe are appropriate. I hope this will not be misinterpreted by the authors and they can take it as a guide.
Additionally, the authors should review the journal format and make it consistent.

TITLE
The title would be more correct in the following way:

Impact of severe mental illnesses on health-related quality of life among patients attending the Institute of Psychiatry, Rawalpindi from 2019 to 2021: a cross-sectional study.


ABSTRACT

This section should be rewritten according to the recommendations I will make throughout the paper.
Keywords should not be acronyms.

INTRODUCTION
The introduction provides a comprehensive overview of the global burden of severe mental illnesses, their impact on health-related quality of life, and the use of assessment instruments to determine the effect of various treatment modalities. It also highlights the need to study health-related quality of life in patients with severe mental illnesses in the local settings and using the EQ-5D-5L instrument. To further strengthen the introduction, it may be useful to include information on the prevalence of severe mental illnesses in Pakistan, particularly in the study location of Rawalpindi, and how this compares to global figures.

On the other hand, in terms of writing, it is very difficult to read, in addition to spelling errors. What is the reason for the authors not listing the references in order?
My suggestion for the wording is as follows.

First paragraph

I would phrase it in this way.

“Severe mental illnesses are associated with a significant global disease burden, with nearly 254 million people suffering from depression, 45 million from bipolar affective disorder, and 20 million from schizophrenia, according to the World Health Organization. These illnesses result in a significant amount of morbidity, measured in disability-adjusted life years (DALYs), and are associated with shorter life expectancies and increased risk of early mortality.”

Second paragraph

I would phrase it in this way.

“Hence quality of life is a broader concept, involving not only the subjective burden but also includes all aspects of daily living, interaction with others. Recent studies have shown an increase in research on health-related quality of life, highlighting its significance as a primary patient outcome.”

Third paragraph

I would phrase it in this way.

“Quality of life in patients suffering from severe mental illness is lower compared to the general population. The impact on a patient's health-related quality of life is influenced by a combination of clinical factors, environmental factors such as social support, and demographic factors [3]. In a cross sectional survey conducted in Germany in 2020, which included various severe mental illnesses such as dementia in addition to schizophrenia, major depressive disorder, anxiety disorders and neurotic disorders, revealed a lower health-related quality of life across all conditions. The study identified demographic factors such as age, lack of social support, and income as major predictors of quality of life impairment [15]. Another study carried out in 2018, examining health-related quality of life in schizophrenia identified additional factors such as clinical symptoms and the presence of comorbidities [13].”

Fourth paragraph

I would phrase it in this way.

“Recently, health-related quality of life assessment has become a routine practice for evaluating the impact of many psychiatric and physical disorders, as well as neurocognitive disorders such as Alzheimer's dementia [4]. This assessment is also used to evaluate various psychiatric disorders including obsessive-compulsive disorder, eating disorders, and various psychotic illnesses. Additionally, it is used to determine the effectiveness of different treatment modalities for these illnesses [5-7].”

Fifth paragraph

I would phrase it in this way.

“There are various instruments available to measure health-related quality of life in patients suffering from various psychiatric illnesses, depending on the patient's clinical characteristics and sociocultural factors, including language. International studies have used EQ-5D-5L to determine the burden of severe mental illnesses at both primary and tertiary care levels [8-12]. However, there is limited evidence of studies that have used EQ-5D-5L to measure health-related quality of life in patients with severe mental illnesses in our local settings. This study aims to use this instrument to determine health-related quality of life in our local tertiary care setting for people suffering from severe mental illnesses, in order to highlight their burden and determine the major sociodemographic factors that affect health-related quality of life.”

Whole of the introduction revised as suggested by the reviewer .


METHODS

Study Design

I would phrase:

“This was a descriptive cross-sectional study, using retrospective record view of data.”

Study Site

I would phrase it in this way.

“The study was conducted at the outpatient and inpatient units of the Institute of Psychiatry located at Benazir Bhutto Hospital in Rawalpindi, which is one of the major mental health facilities in northern Punjab. It is affiliated with Rawalpindi Medical University and is involved in teaching and training of postgraduate residents. The hospital has a 40-bed indoor facility and an outpatient facility with a patient flow of 200 patients per day. The study was conducted as part of the IMPACT (Improving Physical and Mental Health Together) program at the Department of Research and Development, Institute of Psychiatry, Rawalpindi.”

The information provided seems sufficient to understand the study site and its context. It mentions the name of the hospital and the university it is affiliated with, the types of units (outpatient and inpatient), the capacity of the indoor facility, the patient flow of the outpatient facility, and the fact that the study was conducted under a specific program at the department of Research and Development.

Study population

I would phrase it in this way.
“All patients diagnosed with severe mental illnesses (depression with psychotic features, bipolar affective disorder, and schizophrenia) according to the ICD-10 criteria [13], who were 18 years of age or older and enrolled at the study site between July 2019 and March 2021, were included in the study.”

It provides sufficient information about the study population.


I would divide the section "Data variables, source of data and data collection" into two different ones: Data collection method and Variables and sources of data

Data collection method

Qualtrics survey would be the data collection method


Variables and sources of data

I would phrase it in this way.

“First, SMI patients were diagnosed using the MINI neuro psychiatric interview, and then demographic data was collected using the WHO STEPS module for age, gender, income, and educational status. The primary outcome variable was health-related quality of life, which was assessed using the EQ-5D-5L questionnaire, consisting of domains such as mobility, self-care, usual activities, pain and discomfort, and anxiety and depression. The EQ-5D-5L also included a visual analogue scale (EQ-5D-VAS) to allow patients to subjectively evaluate their health state on a scale of 0 (worst imaginable health state) to 100 (best imaginable health state).”

A clearer presentation would be achieved if the authors divided the variables into independent variables (clinical and sociodemographic), and dependent variable/s.

Whole methodology revised as suggested by the reviewer

Analysis and statistics

I would phrase it in this way.
“The data was entered into SPSS version 26, and the key analytical outputs included the level of impairment in health-related quality of life in patients with bipolar affective disorder, schizophrenia, and depression with psychotic features. Descriptive data analysis was used to assess the proportions of patients in each domain of the quality-of-life questionnaire, and associations with various sociodemographic factors were determined using a chi-square test. A p-value of <0.05 was considered statistically significant.”

The data analysis described appears to be appropriate for the study. Descriptive analysis can help provide a better understanding of the patient population and determine the proportions of patients in each domain of the quality-of-life questionnaire. The chi-square test can help identify associations between sociodemographic factors and quality-of-life outcomes in patients with severe mental illness.

Whole analysis section rewritten as suggested by the reviewer

Ethical considerations

I would phrase it in this way.

“The institutional research committee at Rawalpindi Medical University was contacted for permission to use data on individuals with SMI. The data was entered into a pre-designed format based on information from the database, and personal identifying information and patient names were excluded from the analysis and final presentation of study findings. Hard copies of the data were kept under lock and key, while electronic files were password protected. Access to the data for analysis and interpretation of results was limited to authorized persons, such as the principal investigator.”

Ethical considerations also rewritten as suggested by the reviewer


RESULTS

I would phrase it in this way.

“The study included 922 SMI patients, of whom 555 (60.2%) were male and 367 (39.69%) were female. The participants suffered from major depressive disorder (422; 45.8%), bipolar affective disorder (392; 42.51%), and schizophrenia (108; 11.7%). The majority of participants were in the younger age group, with 80% of the population being below 50 years old, and had an education level below secondary education (57.4%). A detailed breakdown of the demographics can be found in Table 1.

In the analysis of associations between EQ-VAS (subjective quality of life scale) and demographic factors, a significant association was found with gender (p=0.000) and education (p=0.000). Women had lower EQ-VAS scores (49.43±27.72) compared to men (58.81±27.1), and individuals with lower education also had lower mean scores. Details can be found in Table 2.

The analysis of EQ-VAS scores across severe mental illnesses (SMI) revealed that patients with depression had lower scores across all demographic factors compared to patients with schizophrenia and bipolar affective disorder, as shown in Table 3.

When asked about their health-related quality of life, most participants who were suffering from depression reported experiencing problems with mobility (73%), self-care (62%), activities (70%), pain (87.5%), and anxiety/depression (90%), with severity ranging from mild to severe. This indicates that the health-related quality of life of participants with depression was lower compared to those with bipolar affective disorder and schizophrenia, as demonstrated in Table 4.”

Setting aside the issue of wording, this section provides a succinct summary of the study's participants and key findings, with references to the relevant tables.
To enhance comprehension, it is advised that the authors standardize the format and improve the presentation of the tables.

Results section rephrased as suggested by the reviewer

DISCUSSION

I would phrase it in this way.

“Our study has determined that the health-related quality of life, a major outcome for patients, was decreased in all populations with severe mental illness (SMI). However, patients suffering from depression showed more significant results with lower mean EQ-VAS scores compared to those with schizophrenia and bipolar disorder. Demographic factors, such as gender and education, were found to have a significant association with health-related quality of life. Females had lower scores (49.43 ± 27) than males (58.81 ± 27), and people with lower education levels reported lower scores. Objectively assessing health-related quality of life in all domains of EQ-5D-5L, patients with depression reported more problems with mobility (73%), self-care (62%), usual daily activities (70%), perception of pain (87.5%), and symptoms of anxiety/depression (90%), ranging from mild to severe in intensity, compared to patients with bipolar disorder and schizophrenia. To improve overall health-related quality of life for SMI populations, targeted interventions for patients with depression should be considered.

Our study revealed that severe mental illness patients have a generally low health-related quality of life. Various other studies have been conducted to assess the health-related quality of life in this population, all of which indicate a lower quality of life in these patients. For example, a 2021 study determined that patients with bipolar affective disorder had poor health in both physical and mental health parameters, which is consistent with the lower health-related quality of life found in this SMI population [14]. Similar findings have been reported in other studies, which are comparable to the results of our study. ¿References?

Our study findings indicate that patients with depression have a lower health-related quality of life than those with other severe mental illnesses, which is consistent with the results of a study conducted in 2020 [15]. The decreased quality of life in depression patients could be due to the clinical features of depression, such as physical symptoms like fatigue, insomnia, and decreased appetite, as well as emotional and cognitive symptoms like concentration and memory problems. Studies have also shown that the severity of symptoms, number of episodes, and residual symptoms can impair the health-related quality of life in these patients [17]. Additionally, patients with depression may have better insight into their illness and be more aware of its impact on their quality of life compared to those with bipolar affective disorder, particularly with psychotic features, and schizophrenia.

The low socioeconomic status of the majority of the population in low- and middle-income countries, such as Pakistan, results in poor access to mental health facilities and inadequate treatment, leading to lower health-related quality of life. Therefore, the factor of poor socioeconomic status becomes more relevant in contributing to the lower health-related quality of life in our population. Additionally, lack of awareness about mental illness due to low educational status regarding disease course, prognosis, and proper treatment and follow-ups can be a cause of its association with lower health-related quality of life. These findings are consistent with a 2020 study that identified age, income, and CGI scores as potential influencers on health-related quality of life, which is also consistent with the demographic factors identified in our study, such as education, gender, and age [15].

A study conducted under the STAR*D trial revealed that demographic factors such as low education, poor socioeconomic status, and lack of social support were associated with lower health-related quality of life in depression. Additionally, individuals who were widowed, divorced, or separated also exhibited lowered health-related quality of life [17].

The lower health-related quality of life in females may be attributed to several factors. Firstly, studies have reported lower health-related quality of life in the general female population [18]. Additionally, in low- and middle-income countries such as Pakistan, females typically have lower levels of education and socioeconomic status than males, making these factors more relevant in contributing to the lower health-related quality of life in this population. Stigmatization may also play a role in this disparity.”

Similarly, in the results section, setting aside the issue of wording, the discussion presents the study's key findings and discusses the significance of these findings in the context of previous research. It also identifies demographic factors that are associated with lower health-related quality of life in patients with severe mental illness, such as gender and education. The discussion also highlights the importance of targeted interventions for patients with depression and the need to address socioeconomic factors that contribute to lower health-related quality of life in low- and middle-income countries. Overall, the discussion provides a comprehensive summary of the study's findings and their implications.

The authors should include sections such as strengths and limitations of the study.

Strengths and limitations of the study

"Our study is among the few conducted in Pakistan that examined the impact of severe mental illnesses on health-related quality of life using the standard Eq-5d-5l questionnaire. It is also unique in identifying the sociodemographic factors that affect health-related quality of life in a diverse population of severe mental illness patients in a low- and middle-income country like Pakistan.

However, we did not examine specific clinical features such as the nature and severity of symptoms and comorbidities, while other studies, such as one conducted in the United States, have identified clinical factors that contribute to health-related quality of life impairment in patients with schizophrenia. These factors include positive and negative symptoms of schizophrenia. [16]

Another limitation was due to its correlational design, as it only allowed us to explore associations between demographic factors and health-related quality of life, without establishing any causal relationships."

Limitations that should be added:

Study that relies on retrospective record view of data is the possibility of incomplete or inaccurate data. Since the data is being gathered from past records, there is a chance that some information may be missing or incorrect.
In addition, the study design may not allow for exploration of complex relationships or underlying factors that may influence the data, as it is limited to a descriptive analysis of the available information.

Broader strength and limitations added in the manuscript as suggested by the reviewer

The authors should include a Conclusion section.


CONCLUSIONS

I would phrase it in this way.

“Our findings suggest that health-related quality of life should be considered an important outcome measure in the management of SMI patients. Regular assessment of health-related quality of life using both objective and subjective measures should be incorporated in routine follow-up visits. This could lead to the development of person-centered care plans that address the specific needs of individual patients, such as improved access to healthcare and social services. This approach may result in better outcomes for SMI patients, as compared to the traditional clinician-oriented approach.”

Conclusion section modified as suggested by the reviewer

The references are not uniformly written.

References modified as suggested by the reviewer.
