# Supplementary material for: Impact of severe mental illnesses on health-related quality of life among patients attending the Institute of Psychiatry, Rawalpindi from 2019 to 2021: A cross-sectional study
Source: PLoS One. 2023 Aug 3;18(8):e0289080. doi: 10.1371/journal.pone.0289080 (PMC10399870; doi:10.1371/journal.pone.0289080)
Supplement: Attachment — Submitted filename: new REBUTTAL LETTER.docx [file pone.0289080.s004.docx]

**REBUTTAL LETTER**

**Thank you for all reviewers for their useful comments and suggestions, made for improving the original manuscript. we hope our attempt to deal with reviewer’s concerns is considered satisfactory.**

**For a better understanding we have colored all the clarifications in red**

PONE-D-23-01479R1

Impact of severe mental illnesses on health-related quality of life among patients attending the Institute of Psychiatry, Rawalpindi from 2019 to 2021: a cross-sectional study

Specific comments:
Introduction
“Recent studies have shown an increase in research on health-related quality of life, highlighting its significance as a primary patient outcome” – I think this sentence needs backing up, with reference to at least some recent studies or a review of recent studies.

Reference was not added because in the following paragraphs there is detail of all studies with references which have used health related quality of life as an outcome measure

The numbering of references is still not in numerical order, with references running 1, 2, 3, 15, 16 in the introduction.

Numbering of references corrected in whole of manuscript as suggested by the reviewer.

Methods
EQ-5D-5L is spelled out in the text on the second use, rather than the first (two lines above where it is spelled out).
Could the authors add that the questionnaire was self-completed in the text for clarity?

Added as suggested by reviewer.

Results
While you’ve added the p-value to the marital status in the abstract, it isn’t added to the second paragraph of the results – “p<0.001)” just needs inserting after “marital status”.

P value added to marital status in the results as suggested by the reviewer

“Women had lower EQ-VAS scores (49.43±27.72) as compared to males (58.81±27.1)”: This needs labelling as (presumably) mean and standard deviation.

Mean and standard deviation labelled as suggested by the reviewer.

Table 1: Thank you for adding the results by diagnosis. I think the percentages for the total SMI patients should probably be the percentage in each group – so male: 555 (60.2), female (39.8) rather than 100 for all.

Percentages added in table no 1 as suggested by the reviewer.

Table 2: For consistency/clarity I’d suggest reporting p-values as <0.001 rather than 0.000.

p values added as suggested by the reviewer in table no 2

Table 2: Typo in the heading “DEMOGRPAHIC”

Corrected

Discussion
“Our study has determined that health related quality of life, a major patient outcome was decreased in all population with SMI (severe mental illness).” I am still unsure about this statement – decreased compared to what? Perhaps “Our study has determined that health related quality of life, a major patient outcome was low, in all population with SMI”.

Sentence formatted as suggested by the reviewer in the discussion
